# Inhibition of MEK/ERK signalling pathway promotes erythroid differentiation and reduces HSCs engraftment in *ex vivo* expanded haematopoietic stem cells

**DOI:** 10.1111/jcmm.13379

**Published:** 2017-10-10

**Authors:** Morteza Zarrabi, Elaheh Afzal, Mohammad Hossein Asghari, Monireh Mohammad, Hamidreza Aboulkheyr Es, Marzieh Ebrahimi

**Affiliations:** ^1^ Department of Stem Cells and Developmental Biology Cell Science Research Center Royan Institute for Stem Cell Biology and Technology, ACECR Tehran Iran; ^2^ Royan Stem Cell Technology Company, Cord Blood Bank Tehran Iran; ^3^ Animal Core Facility Reproductive Biomedicine Research Center Royan Institute for Animal Biotechnology, ACECR Tehran Iran

**Keywords:** MEK/ERK pathway, human umbilical cord blood (hUCB), haematopoietic stem cells (HSCs), *ex vivo* expansion, erythroid differentiation

## Abstract

The MEK/ERK pathway is found to be important in regulating different biological processes such as proliferation, differentiation and survival in a wide variety of cells. However, its role in self‐renewal of haematopoietic stem cells is controversial and remains to be clarified. The aim of this study was to understand the role of MEK/ERK pathway in *ex vivo* expansion of mononuclear cells (MNCs) and purified CD34^+^ cells, both derived from human umbilical cord blood (hUCB). Based on our results, culturing the cells in the presence of an inhibitor of MEK/ERK pathway—PD0325901 (PD)—significantly reduces the expansion of CD34^+^ and CD34^+^ CD38^−^ cells, while there is no change in the expression of stemness‐related genes (*HOXB4, BMI1*). Moreover, *in vivo* analysis demonstrates that PD reduces engraftment capacity of *ex vivo* expanded CD34^+^ cells. Notably, when ERK pathway is blocked in UCB‐MNCs, spontaneous erythroid differentiation is promoted, found in concomitant with increasing number of burst‐forming unit‐erythroid colony (BFU‐E) as well as enhancement of erythroid glycophorin‐A marker. These results are in total conformity with up‐regulation of some erythroid enhancer genes (*TAL1, GATA2, LMO2*) and down‐regulation of some erythroid repressor genes (*JUN, PU1*) as well. Taken together, our results support the idea that MEK/ERK pathway has a critical role in achieving the correct balance between self‐renewal and differentiation of UCB cells. Also, we suggest that inhibition of ERK signalling could likely be a new key for erythroid induction of UCB‐haematopoietic progenitor cells.

## Introduction

Haematopoietic stem cells (HSCs), the most recognized stem cells in the field of cell therapy, have been used in clinic for the last three decades 1. These cells are responsible for populating and sustaining the blood system through highly coordinated self‐renewal and differentiation process 2, 3. So far, extensive efforts have been made to identify the mechanisms controlling self‐renewal, differentiation and homing of HSCs 4. However, the key signalling molecules involved in determining the fate of these cells are not fully understood.

The extracellular signal‐regulated protein kinases 1 and 2 (ERK1/2) belong to the mitogen‐activated protein kinase (MAPK) super family that transmit signals from various cell surface receptors to cytosolic and nuclear targets 5. In a variety of cell types, the activation of RAS/MEK/ERK cascade leads to promoting the cell proliferation and survival 6, 7, 8. However, this is not the case for all cell types. Remarkably, the ERK1/2 signalling is dispensable for proliferation and self‐renewal of embryonic stem cells, whereas there is dependency on ERK upon lineage commitment 9, 10.

In haematopoietic system, *in vivo* analysis of ERK1^−/−^ mice has revealed an essential function of ERK1 through thymocyte maturation 11. In addition, based on *ex vivo* studies, ERK pathway plays a critical role in regulating differentiation of megakaryocyte 12, erythrocyte 13, 14, macrophage 15, as well as granulocyte and monocyte 16, 17. Indeed, it seems that activation of ERK pathway may somehow act as a stimulus for HSCs to exit from the self‐renewal programme and enter into differentiation phase 18. Furthermore, there is more evidence that ERK1/2 signalling pathway may also be involved in regulation of other cellular processes of haematopoietic system 19.

The HSCs fate can be affected by time and duration of ERK activation as well as paracrine stimulations from other cells in developmental milieu. To understand more about the precise role of ERK signalling in HSCs fate determination, we used PD0325901 (PD) to block the MEK/ERK pathway in purified UCB‐CD34^+^ cells and their more commitment progenitors in UCB‐MNCs. The effect of ERK inhibition on cord blood cells was assessed after 10 days in serum‐free liquid cultures containing stem cell factor (SCF), Fms‐like tyrosine kinase 3 ligand (Flt3L) and thrombopoietin (TPO), in which the cells are in active expansion phase through proliferation and self‐renewal (Fig. S1).

Here, we show that ERK1/2 activation is required for the maintenance of HSCs self‐renewal and engraftment capacities. Further, according to our results, ERK inhibition by PD and consequently *JUN* hampering promotes the path of erythroid differentiation of MNCs.

## Materials and methods

### Cell culture

Cells were obtained from UCB samples of consenting mothers. Only cord blood samples were used which do not meet the criteria for banking at Royan Cord Blood Bank. Institutional human research ethics approval was also obtained from Royan institute ethic committee (IR.ACECR.ROYAN.REC.1394.175).

MNCs were isolated using 6% hydroxyethyl starch (HES) followed by LymphoprepTM (Stem cell Technology Inc., Canada) density gradient centrifugation. The purity of CD34^+^ cells was enriched to greater than 85% using CD34 immunomagnetic selection kit (Miltenyi Biotec, Germany). To *ex vivo* expansion of the cells, 10^6^ MNCs or 10^4^ CD34^+^ cells/well were cultured for 10 days in the StemSpanTM medium (Stem cell Technology Inc.) supplemented with 100 ng/ml SCF, 100 ng/ml Flt3L, 50 ng/ml TPO all from R&D Systems. To inhibit MEK1/2 pathway, PD0325901 (stemgent, USA) was added to expansion medium at the concentration of 0.25 μM. Every 3 days, half of the medium was replaced by the freshly prepared expansion medium.

### Immunophenotyping

Phenotypic analysis of *ex vivo* expanded UCB cells was performed using flow cytometry. Cells were blocked with PBS‐BSA 1% and were stained with PE‐conjugated anti‐human CD34 and Percp anti‐human CD38 antibodies (BD Pharmingen, Germany). The negative population was determined using relevant isotype control antibodies. To remove blood cells, ammonium chloride lysing solution was added to stained leucocyte suspension. At least 10^4^ events were acquired on a Partec PAS system (Germany), and data were analysed with FloMax software.

### Colony‐forming assay

Colony‐forming unit (CFU) assays were performed using MethoCult (H4434, Stem Cell Technologies). Briefly, 2 × 10^3^ MNCs or 300 CD34^+^ cells re‐suspended in 1.1 ml MethoCult diluted with IMDM + 2% FBS at a ratio of 1/10 and were plated in 35‐mm bacterial dishes. CFUs were scored 14–16 days following plating at 4× magnification and scored into three categories: granulocyte–monocyte (GM), granulocyte–erythrocyte–macrophage–megakaryocyte (GEMM), burst‐forming unit‐erythroid colony (BFU‐E).

### Gene expression analysis

Cells were collected and preserved at −70°C until RNA extraction. Total RNA was isolated with QIAzol lysis reagent. Integrity and quality of RNA samples were checked using a Nanodrop (ND‐1000) spectrophotometer. 1 μg of the total RNA was subjected to reverse transcription using oligo‐dT and PrimeScript™ 1st strand cDNA kit (Takara, Japan). Transcript levels were determined using the SYBR Green master mix and Corbett Rotor‐Gene 6000. Gene expression level was normalized to the human GAPDH housekeeping gene. Relative quantification of gene expression relative to the positive control group was calculated using the ΔΔCt method. Primer sequences for qRT–PCR are listed in Table S1.

### In‐utero mouse transplantation model

NMRI mice strains (8–10 weeks old) were obtained from the animal facility of Royan Institute. All mice were maintained in accord with guidelines approved by the animal welfare committee of the Royan Institute. Mice were mated under controlled conditions, and formation of vaginal plugs was used to assess pregnancy and gestational age. Pregnant mice were anaesthetized with isoflurane on gestational days 11.5‐13.5, and the uterine horns were exteriorized. Each embryo was injected intraperitoneally with 3–5 × 10^4^ cells in 50 μl PBS. Control animals received only 50 μl PBS. The uterine horns were replaced in the abdomen, followed by abdominal closure. The mothers were kept warm until they recovered from anaesthesia. The ethical committee of Royan Institute approved all procedures (Fig. S2).

### Identification of donor cell engraftment

Newborn mice were expanded with subcutaneous injections of human IL3 (4 ng/g), SCF (4 ng/g) and G‐CSF (50 ng/g), three times a week beginning at 2 weeks of age. For analysing haematopoietic engraftment, 1 week later, the peripheral blood samples of newborn mice were labelled with PE Anti‐Human CD45 (BD Pharmingen™). To delete non‐specific background signal, the cells were stained with the appropriate isotype‐matched control and at least 10^5^ events were acquired on a Partec PAS system. Data were analysed with FloMax software. Moreover, bone marrow smears were prepared, air‐dried and fixed with ice‐cold acetone and subjected for human nuclear antigen staining using specific antibody.

### *In vitro* migration assay

StemSpan medium and 100 ng/ml stromal cell‐derived factor‐1 (SDF‐1; R&D Systems) were placed into the lower chamber of a 24‐well Transwell (Corning, Corning). Freshly isolated or 10‐day expanded CD34^+^ cells (10^5^) in 100 μl medium were loaded into the upper chamber over a porous membrane (pore size, 5 μm). After 4‐hr incubation at 37°C, the upper chamber was removed, and the cells in the bottom were collected and counted. Control experiments were performed without SDF‐1 in the lower chamber.

### Protein–protein interaction analysis

To identify protein–protein interaction (PPI) and gene correlation, Expression2Kinases (X2K) software packages including Gene2Network and Gene2List (Version 1.6.1207, Mount Sinai School of Medicine, http://www.maayanlab.net/X2K) were used. The methods and databases were in compliance with the software protocol. The output results were optimized by means of yED works Software (yWorks GmbH, Germany, version 3.16.2.1). Then, the correlation and interaction between selected proteins were analysed by Pathway common‐PCvis software (http://www.pathwaycommons.org/pcviz). The linked proteins to CD34 in visualized network were mapped in detail in terms of their role in erythroid differentiation by applying protein–protein interaction software STRING (http://string-db.org/).

### Statistical analysis

All the data were presented as mean ± S.D. of at least three different biological replicates. Statistical comparisons between the groups were examined by two‐tailed Student's *t*‐test assuming unequal variances. *P* < 0.05 was considered statistically significant difference.

## Results

### ERK1/2 pathway is critical for *in vitro* proliferation of UCB cells

**Figure 1:**
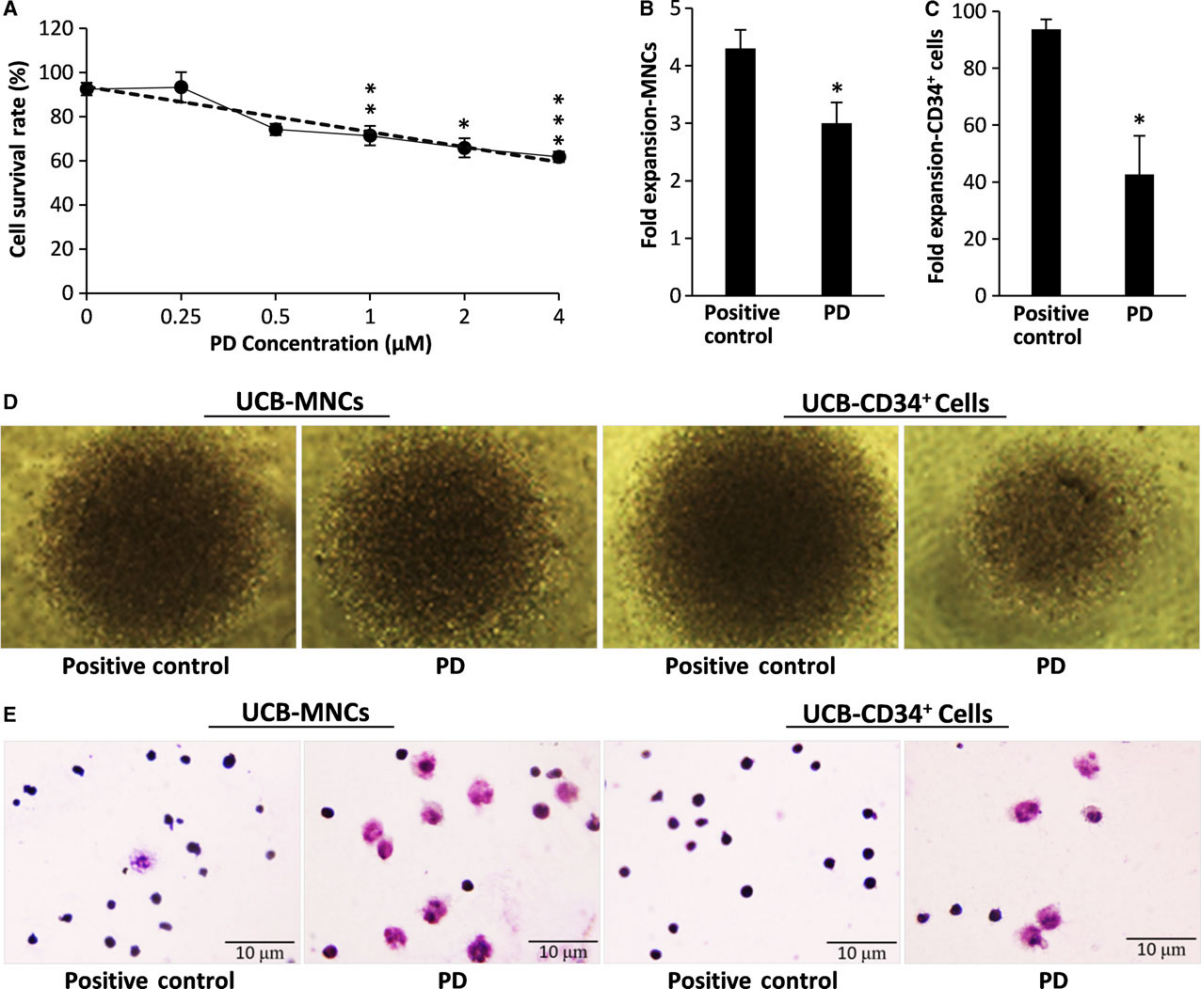
(**A**) UCB‐MNC cells were cultured in cytokine containing medium in the presence of indicated concentrations of PD0325901 for 48 hrs. Then, cell viability was analysed by MTS assay. Results indicated that PD decreased viable cells, dose dependently. (**B**) Effect of PD on the fold expansion of UCB‐MNCs. (**C**) Effect of PD on the fold expansion of UCB‐CD34^+^ cells. Fold expansion was determined by dividing the total number of viable cells after 10 days of culture with cytokines in the presence or absence of PD, by the number of viable cells in the first day of culture. Each bar represents the fold expansion of total nucleated cells (TNCs) per well. *n* = 3, statistically significant difference compared with positive control group, *P ≤ 0.05, **P ≤ 0.01, ***P ≤ 0.001. (**D**) Phase‐contrast microscopy of proliferating cells cultured in 96‐well plate for 10 days (described in **B** and **C**). (**E**) Giemsa staining images of proliferating cells cultured for 10 days (described in **B** and **C**).

To determine whether the MEK/ERK signalling pathway has an essential function in regulating the proliferation process of cord blood cells, an *ex vivo* expansion system was used. UCB‐CD34^+^ cells and UCB‐MNCs were cultured for 10 days in the presence of SCF, TPO and FLT3L (STF medium) to induce cell proliferation. To determine the cytotoxic effect of PD0325901 on UCB cells, the cells were cultured in the presence of different concentrations of PD (0–4 μM) for 48 hrs. As shown in Figure 1A, PD reduced dose‐dependently cell viability. Therefore, we chose the concentration of 0.25 μM that was not toxic for the cells. In the presence of selected dose of PD, the number of UCB‐MNCs and UCB‐CD34^+^ cells significantly reduced (1.4‐fold in UCB‐MNCs, *P* < 0.05; and 2.2‐fold in UCB‐CD34^+^ cells; *P* < 0.01) (Fig. 1B and C). As shown in Figure 1D, although the expansion of CD34^+^ cells was superior as we expected, the modest effect of PD was observed in MNCs, which include the more commitment cells. Moreover, PD‐expanded cells changed morphologically. As seen in Figure 1E, the large cytoplasm with side nucleus suggested the myeloblast differentiation of PD‐expanded MNCs as well as PD‐expanded CD34^+^ cells.

### ERK1/2 pathway tunes self‐renewal and differentiation of UCB cells

**Figure 2:**
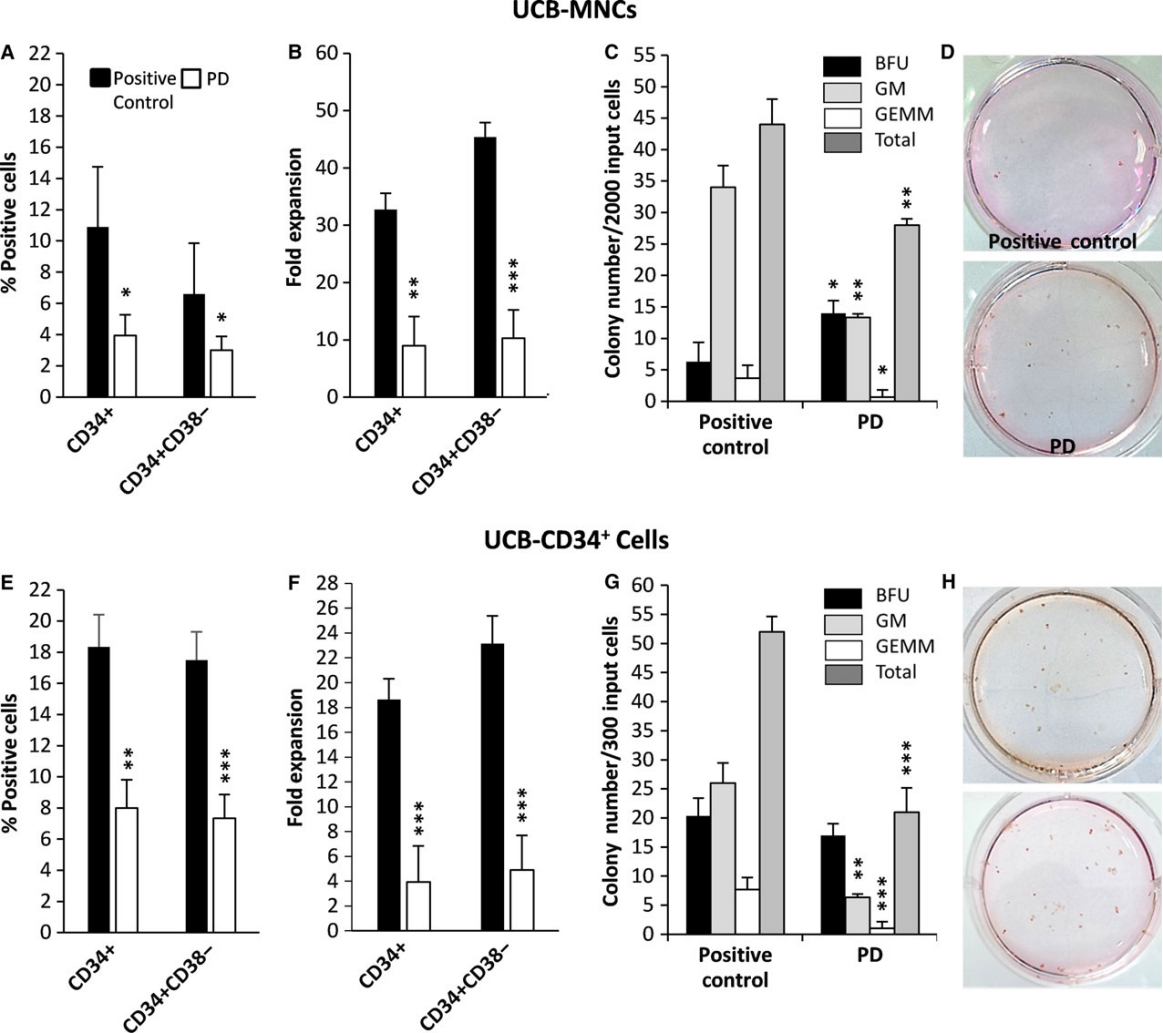
(**A**) The percentage of CD34^+^ and CD34^+^ CD38^−^ cells in UCB‐MNCs and (**E**) UCB‐CD34^+^ cells, expanded for 10 days by cytokines in the presence or absence of PD. (**B**) The fold expansion of CD34^+^ and CD34^+^ CD38^−^ cells in UCB‐MNCs and (**F**) UCB‐CD34^+^ cells expanded for 10 days by cytokines in the presence or absence of PD. Fold expansion was determined by dividing the total number of viable cells expressing the phenotype at the end of the culture by the input number of viable cells expressing the same phenotype (*n* = 3). Classification and number of colony‐forming units (CFUs) in (**C**) 2000 progeny of UCB‐MNCs and (**G**) 300 progeny of UCB‐CD34^+^ cells expanded for 10 days by cytokines in the presence or absence of PD, summarized from three separate samples (*n* = 6), statistically significant difference compared with positive control group, *P ≤ 0.05, **P ≤ 0.01, ***P ≤ 0.001. (**D, H**) Photomicrograph of colonies developed in six‐well plates.

In the next step, we sought the effect of PD0325901 on the self‐renewal and differentiation potential of UCB cells. The percentage of CD34^+^ and CD34^+^ CD38^−^ cells were analysed either in the presence or absence of PD. In first day of experiment, the percentage of CD34^+^ cells was 1.4% in UCB‐MNCs and 90% in UCB‐CD34 purified cells (Fig. S3). Our results showed that under identical culture condition, 10.9 ± 4% of expanded UCB‐MNCs (Fig. 2A) and 18.3 ± 2% of expanded UCB‐CD34^+^ cells (Fig. 2E) were positive for CD34. These numbers significantly declined to 3.9 ± 1.3% and 8 ± 1.8% in the PD‐expanded respective cells. Similarly, cultures containing PD had a lower percentage of CD34^+^ CD38^−^ cells compared with control (6.6 ± 3.6% *versus* 3.0 ± 0.8% in MNCs and 17.5 ± 1.8% *versus* 7.0 ± 1.5% in CD34^+^ cells). Furthermore, the fold expansion of primitive CD34^+^ cells and CD34^+^CD38^‐^ cells analysed in comparison with their initial seeding numbers, confirmed that PD0325901 resulted in huge reduction in numbers of UCB cells either in MNCs or CD34^+^ cells (Fig. 2B and F).

**Figure 3:**
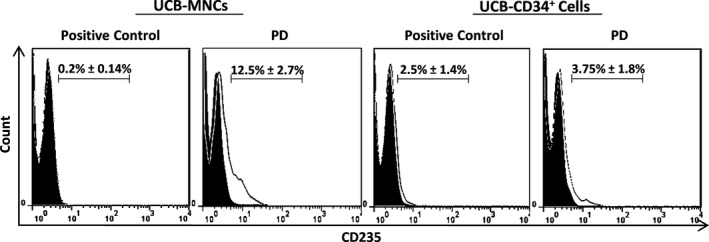
Representative flow cytometric analysis of expanded UCB‐MNCs and UCB‐CD34^+^ cells using monoclonal antibody for glycophorin‐A (CD235). The unfilled curve shows the percentage of CD235 positive cells and the grey filled represents the isotype control.

To assess the differentiation potential of cultured cells, the live cells were plated separately in CFU assays and haematopoietic colonies were counted after 14 days. PD0325901 significantly reduced colony‐forming efficiency either in UCB‐MNCs (Fig. 2C and D) or UCB‐CD34^+^ cells (Fig. 2G and H), mainly in CFU‐GM and CFU‐GEMM. However, the number of erythroid (BFU) colonies was increased, especially in UCB‐MNCs population (Fig. 2C). The increase in the expression of erythroid surface marker glycophorin‐A (CD235) also confirmed our data for promotion of erythroid differentiation in UCB‐MNCs (Fig. 3).

### MEK/ERK inhibition interferes with the UCB cells development

**Figure 4:**
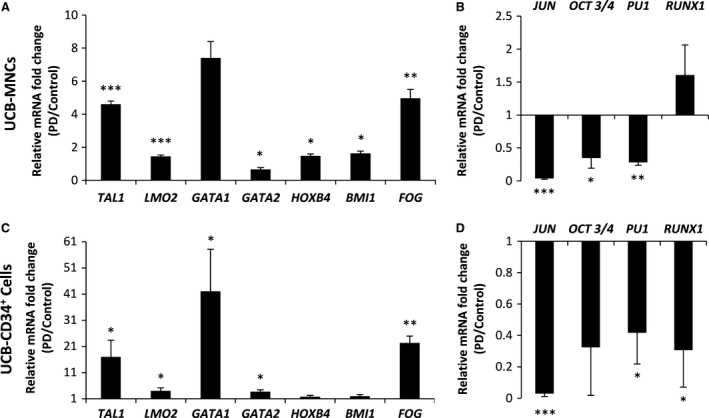
Treatment by PD modifies the gene expression of UCB‐MNCs (**A, B**) and UCB‐CD34^+^ cells (**C, D**). Bars represent the mean fold changes of gene expression in the PD‐expanded cells relative to the positive control group detected by quantitative real‐time PCR (*n* = 3), *P ≤ 0.05, **P ≤ 0.01, ***P ≤ 0.001 *versus* positive control.

The colony assay data revealed that inhibition of ERK pathway results in induction of erythroid differentiation which is dominant in MNCs as well as reduction of myeloid differentiation which is dominant in CD34^+^ cells. Therefore, we checked the key transcription factors involving in HSCs/HPCs development. As shown in Figure 4, the expression levels of *HOXB4* and *BMI1* genes—generally implicated in HSC self‐renewal 20, 21—were not significantly differ in purified CD34^+^ cells after treatment with PD, but significantly up‐regulated in PD‐expanded MNCs (Fig. 4A and C). However, *OCT3/4*—known recently as a gene involving in HSCs self‐renewal 22—was significantly under expressed in PD‐expanded mononuclear and CD34^+^ cells (Fig. 4B and D). Interestingly, *TAL1, GATA1, LMO2* and *FOG*—the core factors of erythroid transcription complex 23—up‐regulated significantly (Fig. 4A and C) in PD‐expanded cells. Conversely, *PU1* and *JUN*—the important repressors of erythroid differentiation programme 24, 25—significantly down‐regulated in PD‐expanded cells. However, *RUNX1* was under expressed in CD34^+^ group and did not change in UCB‐MNCs (Fig. 4B and D).

### Inhibition of MEK/ERK pathway reduces the engraftment of *ex vivo* expanded UCB‐CD34^+^ cells in immunodeficient mouse embryos

**Figure 5:**
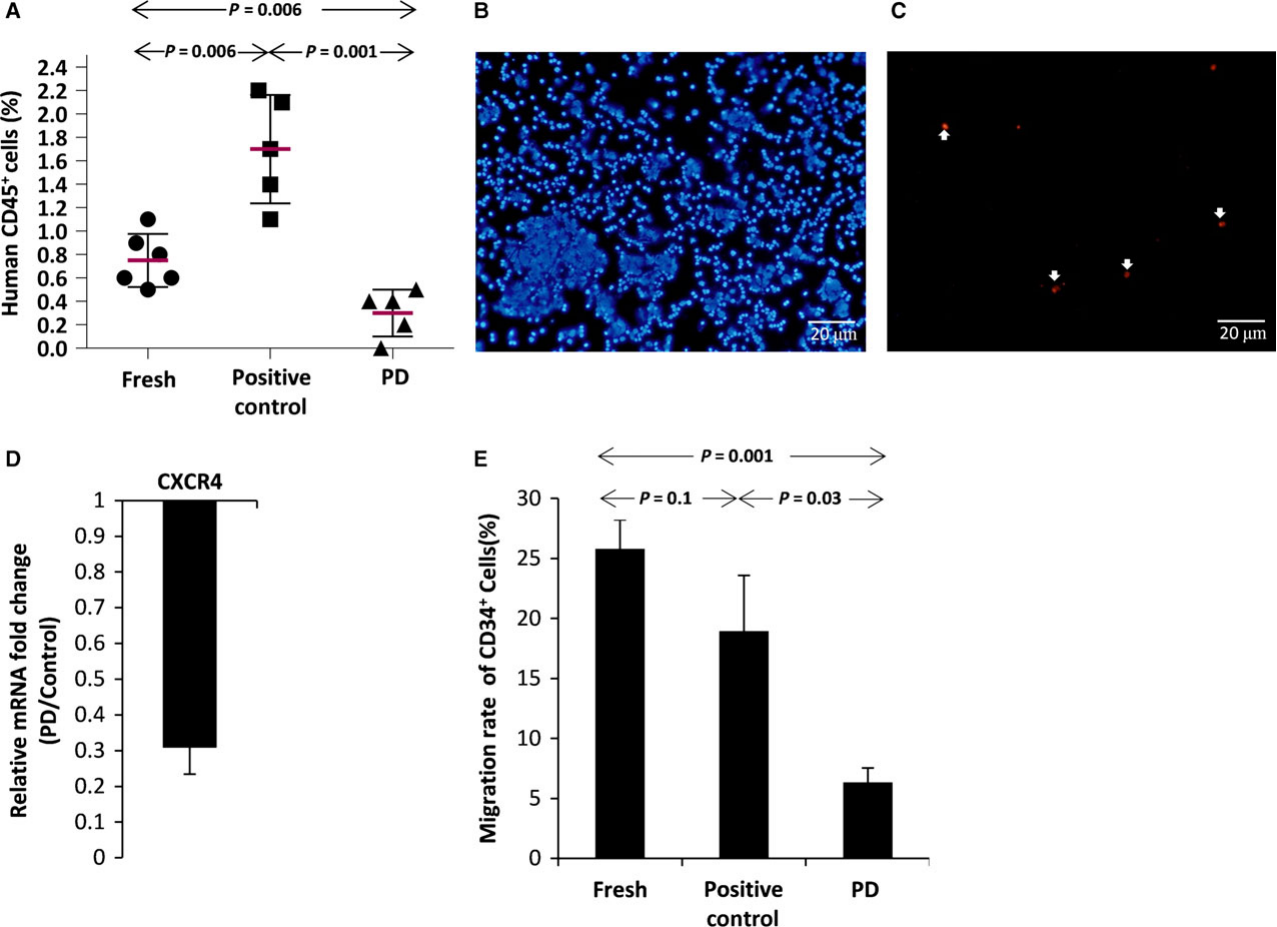
(**A**) Percentage of human CD45 positive cells in peripheral blood of newborn mice after one‐week treatment with human haematopoietic growth factors [SCF (4 ng/g), IL3 (4 ng/g) and G‐CSF (50 ng/g)]. (**B**) Immunocytochemistry to identify human cells in the bone marrow of newborn mice, DAPI stains nucleus (Blue), (**C**) human nuclear antibody (Red). (**D**) CXCR4 gene expression in PD‐expanded CD34^+^ cells relative to the positive control group detected by quantitative real‐time PCR (*n* = 3). (**E**) Percentage of CD34^+^ expanded cells migrated through the transwell in response to SDF‐1 as a chemoattractant factor.

To find the effect of ERK inhibition on engraftment ability of cultured CD34^+^ cells, the short‐term engraftment capacity of fresh and cultured CD34^+^ cells was evaluated through in‐utero transplantation of immunodeficient NMRI embryos 26, 27, 28. Embryos (E11.5–E13.5) were transplanted with 30–50 × 10^3^ primary non‐cultured CD34^+^‏ cells or their entire progeny following 10‐day culture in the presence or absence of PD. Human haematopoietic cell engraftment was assessed in three‐week age mice after one‐week subcutaneous injection of recombinant human growth factors SCF (4 ng/g), IL3 (4 ng/g) and G‐CSF (50 ng/g). As shown in Figure 5A, the average human cell engraftment in peripheral blood of the mice transplanted with freshly isolated CD34^+^ cells was about 0.75 ± 0.22%. The engraftment reduced to less than 0.3 ± 0.2% in mice transplanted with PD‐expanded CD34^+^ cells. In contrast, treatment of CD34^+^ cells with cytokine alone resulted in more than twofold (1.7 ± 0.4%) increase in human cell engraftment compared with the freshly isolated CD34^+^ cells.

Next, to determine the possible cause of poor engraftment ability of PD‐expanded CD34^+^ cells, we examined the effect of MEK/ERK inhibition on *CXCR4* gene expression. The result showed significant down‐regulation of *CXCR4* gene in the cells expanded with PD (Fig. 5D). Moreover, such cells showed less ability to migration towards SDF‐1 medium in comparison with the cells treated only with cytokines (Fig. 5E). So, based on our results, inhibition of MEK/ERK pathway can down‐regulate the *CXCR4* expression in UCB CD34^+^ cells during *ex vivo* expansion, which may also contribute to the reduced engraftment ability of PD‐expanded cells.

## Discussion

The vital role of MEK/ERK signalling in *de novo* generation of foetal hematopoietic stem cells as well as regulation of haematopoietic niche has been revealed before 19, 29. Also, *ex vivo* studies using differentiation‐competent cell lines have shown the importance of ERK signalling in the regulation of myeloid, erythroid, megakaryocyte and thymocyte differentiation 30. However, the precise function of MEK/ERK signalling in *ex vivo* self‐renewal and differentiation of haematopoietic stem cells remains to be understood. To explore this question, we evaluated the function of ERK pathway, during *ex vivo* expansion of purified UCB‐CD34^+^ cells and their more commitment progenitors in UCB‐MNCs. Based on our results, PD could significantly inhibit *ex vivo* proliferation and self‐renewal of both MNCs and CD34^+^ cells. Also, inhibition of ERK signalling by PD leads to spontaneous erythroid differentiation of UCB‐MNCs, which is in concomitant with overexpression of erythroid lineage transcription factors, the presence of more CD235^+^ cells and increasing number of BFU‐E colony in the population of PD‐expanded MNCs. This occurred despite using the effective combination of cytokines (SCF, FLT3L and TPO) to maintain the self‐renewal of HSCs 31. Therefore, it seems that ERK activation may play an important role in achieving the correct balance between the self‐renewal and differentiation of UCB‐MNCs.

*In vivo* studies have shown before that ERK1/2 activity is required for the maintenance of HSCs in a cell‐autonomous manner; and deletion of ERK1/2 leads to leucopenia, anaemia, and early lethality in mice 32, 33. Moreover, promotion of erythroid differentiation upon ERK inhibition has also been observed in avian immature erythroid progenitor cells. Likewise, constitutive activation of the ERK pathway prevents the cells exiting from the self‐renewal and entering a differentiation process 34. Some articles reported that an active form of MEK1 skews HSCs towards the granulocyte/macrophage lineage, specifically during myeloid commitment 16, 35, 36. Also, selective requirement for ERK activation through thymocyte differentiation has been shown before 37, 38. The activation of ERK signalling by GM‐CSF is also considered to be important for differentiation and maturation of macrophage and megakaryocytes 16, 17, 39, 40, 41, 42, 43. Otherwise, it has been reported that survival and proliferation of neutrophil progenitors but not their maturation are dependent to MEK/ERK activation 44. Similarly, ERK1/2 activation is not an essential requirement for growth and differentiation of leukaemic cell lines 45 and even can repress erythroid differentiation of several established cell lines 46, 47. These controversial functions of ERK function may be depend to the source of the target cells “including defined cell lines or purified HSCs derived from bone marrow/cord blood,” the exogenous cytokines in culture medium, the inactivation approach “gene modification or chemical inhibitors,” duration and level of ERK modification 48, 49.

Here, addition of PD0325901 to the culture was associated with increased transcript levels of *GATA1, LMO2, TAL1* and *FOG* which are known to be the key transcription factors for normal erythroid differentiation. These proteins cooperate with each other to form a transcriptional complex which facilitates the GATA1‐mediated development of erythroid cells 50. An interesting question raised for us is how PD can promote erythroid differentiation of UCB‐MNCs. As GATA1 function is required for erythroid differentiation of human haematopoietic stem cells, the c‐Jun ability to repress GATA1 is most likely responsible for inhibition of erythroid differentiation by c‐Jun observed in HSCs 24. On the other hand, c‐Jun has long been recognized as a major downstream target of ERK signalling 51. Here, it seems that inhibition of ERK pathway by PD reversed the inhibition of erythroid differentiation by c‐Jun.

**Figure 6:**
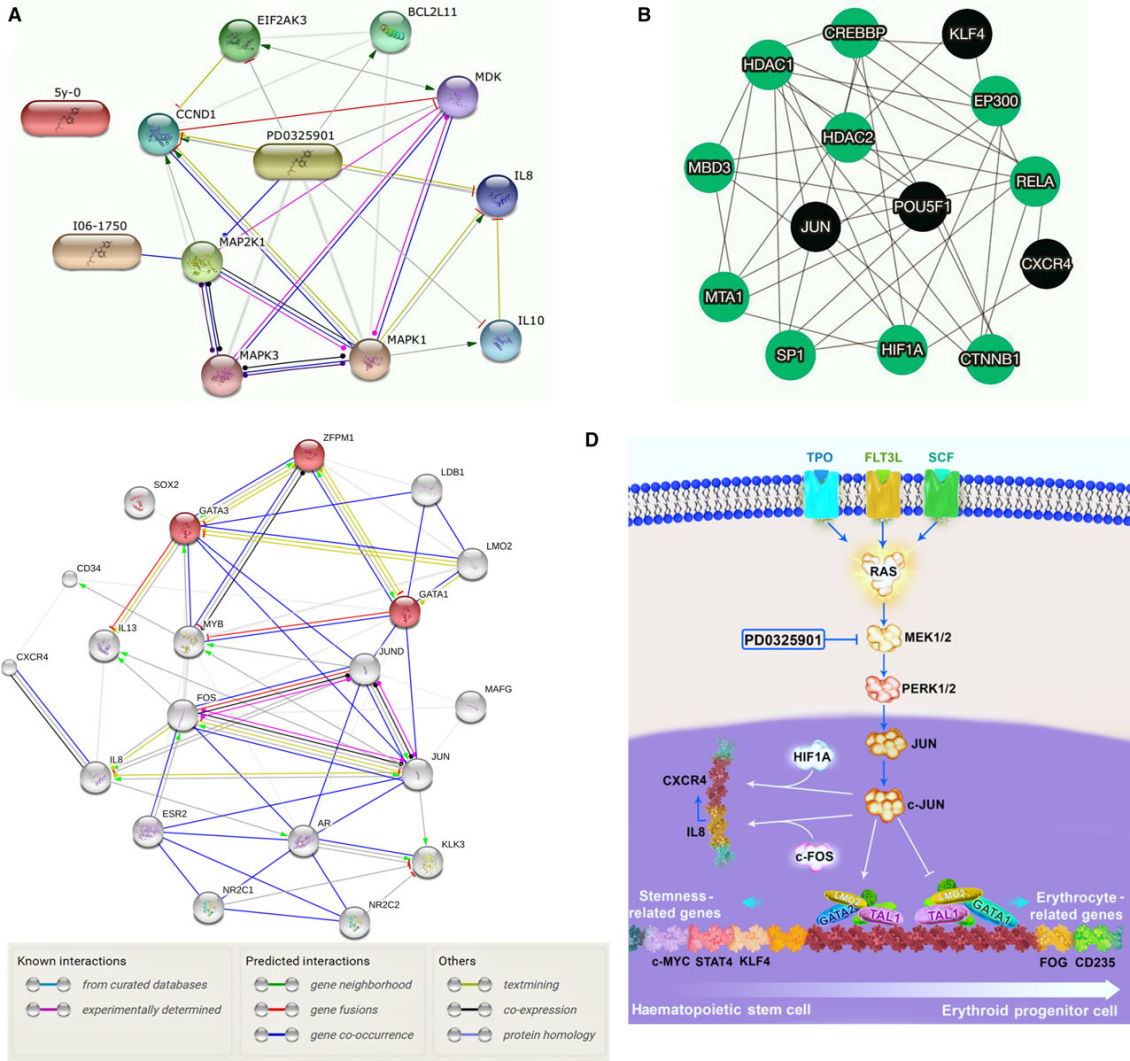
(**A**) String pathway to show the node of PD effects. (**B, C**) Visualization of protein–protein interaction using selected transcription factors in the present study and those which contribute in erythropoiesis. (**D**) Schematic illustration of PD effects on maintenance, differentiation and homing of HSCs.

To further understand the correlation between the transcription factors and the pathways regulated by them, we used data processing software. The PPI network analysis emphasized on the role of c‐Jun and its correlation with other proteins contributed in haematopoiesis (Fig. S4). Unexpected PPI network results showed that *JUN*, indirectly through IL‐8 (Fig 6A) and HIF‐1A (Fig. 6B), plays an important role in the regulation of SDF‐1/CXCR4 signalling (Fig. 6C) 52, 53. It is well known that, the interaction between CXCR4 and SDF‐1 is a key signal, governing the homing of HSCs after transplantation 54. In this study, down‐regulation of CXCR4 gene upon ERK inhibition, may also contribute to the decreased engraftment ability of PD‐expanded CD34^+^ cells. This suggestion is partially supported by the finding that significantly less PD‐expanded CD34^+^ cells migrated in response to SDF‐1 as compared with the cells cultured without PD. Furthermore, *JUN* controls the expression of *KLF4, STAT3* and *c‐MYC* genes which are involved in the cell cycle regulation 55, 56, 57. In summary, based on our results, *JUN* is most likely a key node that controls self‐renewal, differentiation and engraftment of UCB‐haematopoietic stem cells (Fig. 6D).

## Conclusion

Our results showed that the ERK1/2 signalling pathway plays an important role in the maintenance, proliferation and engraftment of UCB‐mononuclear and UCB‐CD34^+^ cells. Furthermore, according to our result, the cross‐talk between HSCs with other commitment cells in UCB‐MNC population, coupled with inhibition of ERK signalling could promote erythroid differentiation of the UCB‐haematopoietic progenitor cells. Also we suggest that erythroid induction of haematopoietic progenitor cells is primarily attributable to PD‐mediated inhibition of Jun activation.

## Conflict of interest

The authors confirm that there are no conflicts of interest.

## Supporting information


**Figure S1** (**A**) Experimental design for investigation of ERk1/2 activity in *ex vivo* expansion of cord blood derived HSCs/PCs.
**Figure S2** In‐utero transplantation of CD34^+^ cells into fetal peritoneal cavity as an immunodeficiency model. The handmade glass micropipette was used to inject cells into fetuses. (**A**) Preparation of the surgery site. (**B**) The uterine horns were exteriorized. (**C**) Each embryo was injected intra‐peritoneally with 30–50 × 10^3^ cells in 50 μl PBS. (**D**) The uterine horns were replaced in the abdomen, followed by abdominal closure.
**Figure S3** Flow cytometry analysis of CD34^+^ cells and CD34^+^ CD38^−^ cells in UCB‐MNCs and UCB‐CD34^+^ cells at the first day of isolation and after 10 days of culture (PD‐expanded cells *versus* positive control group). Just one study selected for data presentation.
**Figure S4** (**A**) Protein‐protein interaction of selected transcription factors obtained in this study and (**B**) suggested correlation by string.
**Table S1** List of primers sequences used in this study
