# Supplementary material for: Inhibition of MEK/ERK signalling pathway promotes erythroid differentiation and reduces HSCs engraftment in ex vivo expanded haematopoietic stem cells
Source: J Cell Mol Med. 2017 Oct 10;22(3):1464–74. doi: 10.1111/jcmm.13379 (PMC5824365; doi:10.1111/jcmm.13379)
Supplement: Supplementary file 1 — Figure S1 (A) Experimental design for investigation of ERk1/2 activity in ex vivo expansion of cord blood derived HSCs/PCs. Figure S2 In‐utero transplantation of CD34+ cells into fetal peritoneal cavity as an immunodeficiency model. The handmade glass micropipette was used to inject cells into fetuses. (A) Preparation of the surgery site. (B) The uterine horns were exteriorized. (C) Each embryo was injected intra‐peritoneally with 30–50 × 103 cells in 50 μl PBS. (D) The uterine horns were replaced in the abdomen, followed by abdominal closure. Figure S3 Flow cytometry analysis of CD34+ cells and CD34+ CD38− cells in UCB‐MNCs and UCB‐CD34+ cells at the first day of isolation and after 10 days of culture (PD‐expanded cells versus positive control group). Just one study selected for data presentation. Figure S4 (A) Protein‐protein interaction of selected transcription factors obtained in this study and (B) suggested correlation by string. Table S1 List of primers sequences used in this study [file JCMM-22-1464-s001.docx]

**Supporting Information**


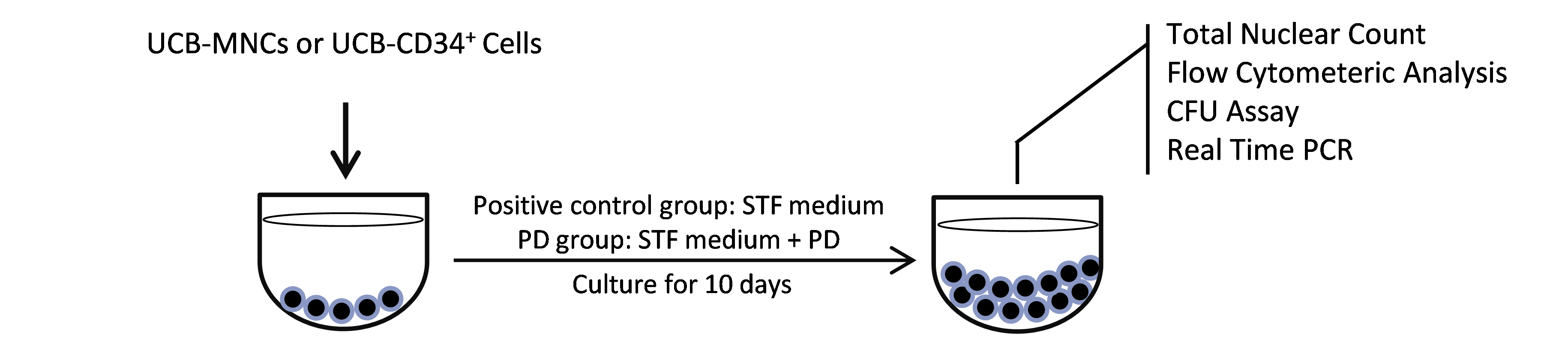


**Figure S1. (A)** Experimental design for investigation of ERk1/2 activity in ex vivo expansion of cord blood derived HSCs/PCs.


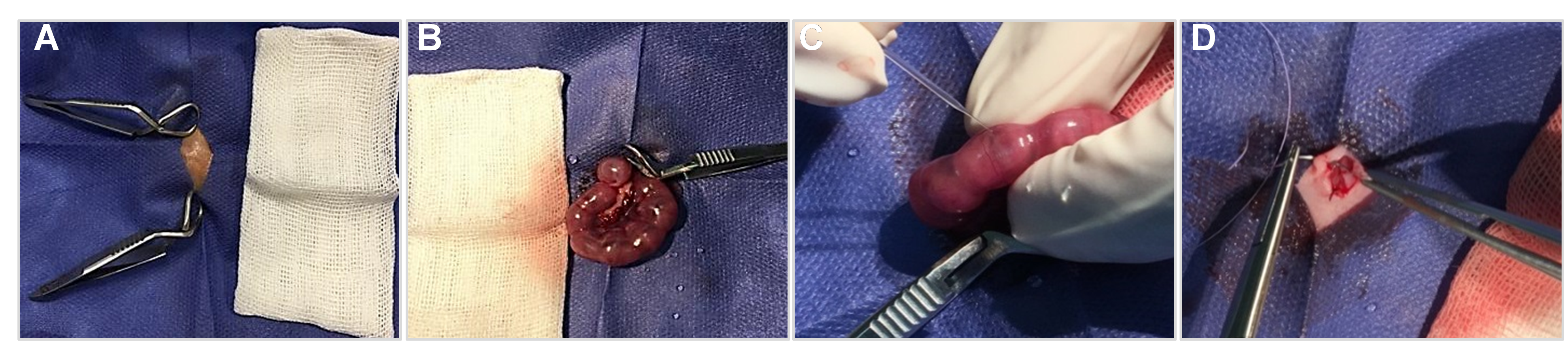


**Figure S2.** In-utero transplantation of CD34^+^ cells into fetal peritoneal cavity as an immunodeficiency model. The handmade glass micropipette was used to inject cells into fetuses. **(A)** Preparation of the surgery site. **(B)** The uterine horns were exteriorized. **(C)** Each embryo was injected intra-peritoneally with 30-50×10^3^ cells in 50μl PBS. **(D)** The uterine horns were replaced in the abdomen, followed by abdominal closure.


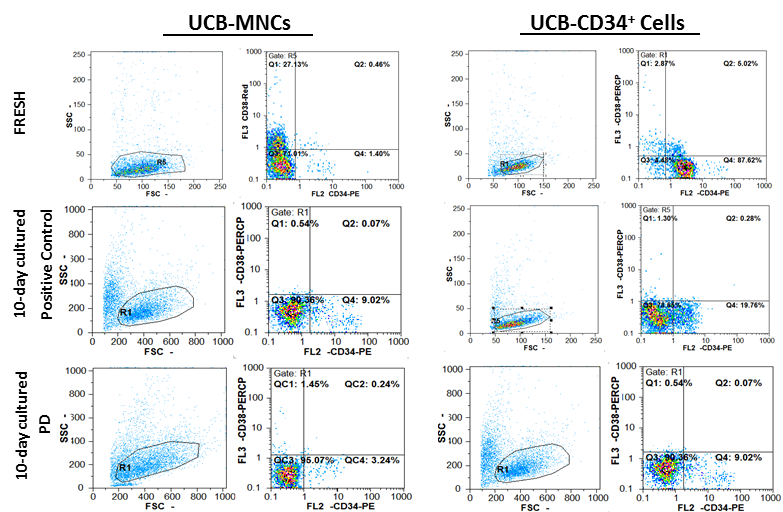


**Figure S3.** Flowcytometry analysis of CD34^+^ cells and CD34^+^CD38^-^ cells in UCB-MNCs and UCB-CD34^+^ Cells from the first day of isolation until 10 days of culture (PD-expanded cells *vs.* control group). Just one study selected for data presentation.


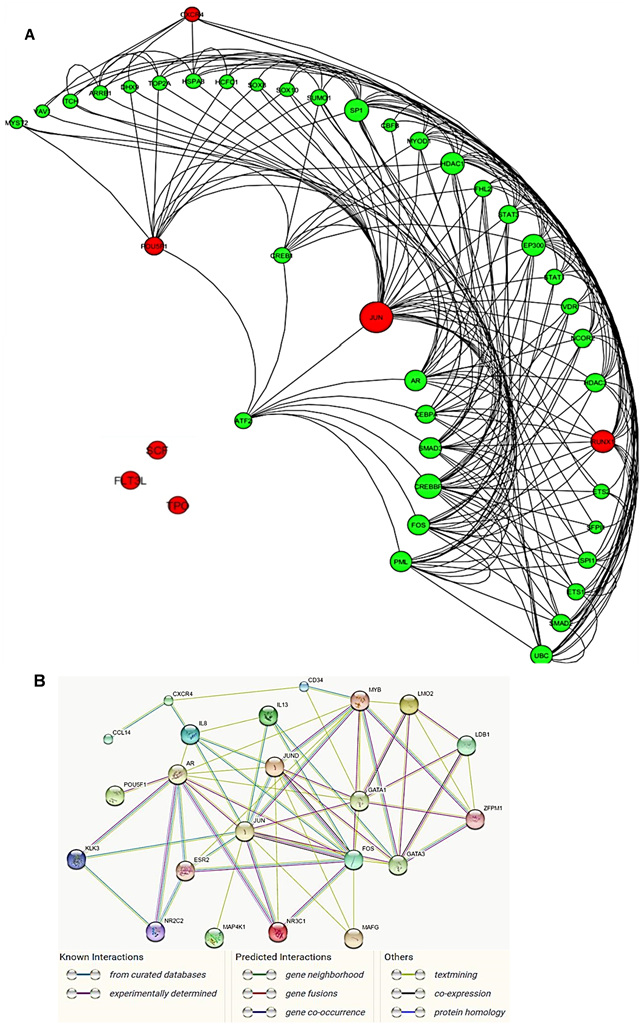


**Figure S4.** **A)** Protein-protein interaction of selected transcription factors obtained in this study and **B)** suggested correlation by string.

**Table S1:** List of primer sequences used in the present study.
